# Disseminated intravascular coagulation in chimeric antigen receptor T-cell therapy: a 6-year nationwide analysis of clinical outcomes and health care resource utilization in patients with hematologic malignancies

**DOI:** 10.1016/j.rpth.2026.106886

**Published:** 2026-07-28

**Authors:** Adamsegd Isac Gebremedhen, Abdu Mohammad, Samhitha Gundakaram, Semere Tesfamariam, Reesha Bodiwala, Mamdouh Souleymane, Muhammad Jamil

**Affiliations:** 1 Department of Internal Medicine, Marshall University School of Medicine, West Virginia, USA; 2 Department of Internal Medicine, Trinity Health, Ohio, USA; 3 Department of Internal Medicine, Emory University School of Medicine, Georgia, USA; 4 Department of Internal Medicine and Pediatrics, Marshall University School of Medicine, West Virginia, USA; 5 Division of Hematology and Oncology, Department of Internal Medicine, Marshall University School of Medicine, West Virginia, USA

**Keywords:** CAR T-cell therapy, disseminated intravascular coagulation, hematologic malignancy

## Abstract

**Background:**

Disseminated intravascular coagulation (DIC) is a recognized complication of chimeric antigen receptor (CAR) T-cell therapy and contributes to treatment-related morbidity and mortality. However, data on its clinical and financial impact remain limited.

**Objectives:**

This study examined the characteristics and outcomes associated with DIC among hospitalizations for hematologic malignancies undergoing CAR-T cell therapy using national U.S. data.

**Methods:**

Adult hospitalizations with acute lymphoblastic leukemia, non-Hodgkin lymphoma, or multiple myeloma who received CAR T-cell therapy (2017-2022) were identified from the National Inpatient Sample. Hospitalizations were stratified by DIC status, and survey-weighted analyses were performed to generate national estimates. Multivariable regression was used to assess differences in mortality and other outcomes (*P* < .05).

**Results:**

Among 10,450 weighted CAR T-cell therapy hospitalizations, 220 (2.1%) developed DIC. In-hospital mortality was significantly higher in hospitalizations with DIC (22.7% vs 2.4%; adjusted odds ratio [aOR], 10.96; 95% confidence interval [CI], 4.78-25.09). DIC was associated with higher odds of acute kidney injury (aOR, 6.01), respiratory failure (aOR, 9.96), and shock (aOR, 18.55) and greater use of renal replacement therapy, mechanical ventilation, and vasopressors. DIC was also associated with higher odds of gastrointestinal hemorrhage (aOR, 12.41) and greater transfusion needs. DIC was associated with longer hospital stays (β = +17.6 days; *P* < .001) and higher hospitalization charges (β = +$523,323; *P* = .02).

**Conclusions:**

DIC is an infrequent yet severe complication of CAR T-cell therapy, associated with increased mortality, organ dysfunction, critical care utilization, transfusion needs, prolonged hospitalization, and higher charges. These findings underscore the importance of clinical vigilance and early intervention strategies.

## Essentials

• Disseminated intravascular coagulation (DIC) is an uncommon but life-threatening complication of CAR T-cell therapy.
• This study examined the characteristics and outcomes associated with DIC among hospitalizations for hematologic malignancies undergoing CAR-T cell therapy using 6 years of national U.S. data (2017-2022).
• Patients with DIC more frequently had low-income status, self-pay insurance, and concomitant sepsis.
• DIC was strongly associated with increased mortality, organ dysfunction, intensive care utilization, transfusion requirements, prolonged hospitalization, and higher health care charges.

## 1 Introduction

Coagulopathy is an increasingly recognized and potentially life-threatening complication following chimeric antigen receptor (CAR) T-cell therapy. The spectrum of hemostatic abnormalities observed in this setting ranges from mild, asymptomatic laboratory findings, such as isolated prolongation of the activated partial thromboplastin time, to severe coagulopathies, including DIC [1,2]. These coagulation disorders frequently emerge in the context of cytokine release syndrome (CRS), a well-characterized and common toxicity associated with CAR T-cell therapy. This association suggests an underlying pathophysiological link mediated by systemic inflammation, endothelial activation, and consumption of coagulation factors [3,4].

Coagulopathy typically develops within a few days of CRS onset and can occur as early as 6 days postinfusion [5,6]. Among the coagulopathies observed, DIC, although relatively rare, represents 1 of the most clinically significant and potentially fatal complications. It has been reported in ∼14% to 50% of patients who develop severe CAR T-cell–associated coagulopathy [7]. The development of DIC appears to correlate strongly with the severity of CRS. In 1 study, DIC was observed in 7 patients (7%) who experienced grade ≥3 CRS. Of these, 2 patients had grade 3 CRS, 2 had grade 4 CRS, and 3 developed grade 5 CRS, highlighting a direct relationship between increasing CRS severity and DIC incidence. Importantly, this association was found to be independent of other clinical variables such as patient age, sex, underlying malignancy, tumor burden, or the type of lymphodepleting chemotherapy administered [5].

The clinical manifestations of DIC in CAR T-cell therapy recipients range from subclinical laboratory derangements to overt hemorrhage, multiorgan dysfunction, and death [5,8]. Data from the US Food and Drug Administration’s Adverse Event Reporting System, analyzing CAR T-cell therapy–related hematologic toxicities from 2017 to 2021, reported DIC-associated mortality rates as high as 59.6% [9].

Despite its potential severity, DIC remains undercharacterized in the context of CAR T-cell therapy, and given the expanding use of CAR T-cell therapies across hematologic malignancies, a deeper understanding of the epidemiology, clinical course, and health care burden of DIC is urgently needed. The present study aims to address this gap by evaluating the incidence, outcomes, and resource utilization associated with DIC in CAR T-cell therapy recipients using nationally representative US data. These findings are expected to inform clinical decision-making and contribute to ongoing efforts to optimize safety and outcomes in patients undergoing CAR T-cell therapy.

## 1 Methods

### 2.1 Data source

We conducted a retrospective analysis of data from 2017 to 2022 using the National Inpatient Sample (NIS). The NIS, a core component of the Healthcare Cost and Utilization Project (HCUP) sponsored by the Agency for Healthcare Research and Quality (AHRQ), is the largest publicly available all-payer inpatient database in the United States. It is designed to generate nationally and regionally representative estimates of hospitalizations, health care utilization, clinical outcomes, and associated costs. It provides detailed discharge-level data, including patient demographics, primary payer, hospital characteristics, diagnoses, and procedures. The NIS design uses a stratified, probability sampling approach based on hospital ownership, bed size, teaching status, US census region, and urban-rural location. A 20% sample of hospitals is drawn from each stratum, and all discharges from sampled hospitals are included. Sampling weights are applied to produce nationally representative estimates. Data are derived from state-level HCUP partners, with recent editions incorporating submissions from up to 48 states and the District of Columbia, representing >97% of the US population. The dataset is limited to community hospitals, defined as non-federal, short-term, general, and specialty facilities. This robust sampling methodology enables comprehensive, population-based analyses of inpatient care in the United States. Methodological details are available at https://www.hcup-us.ahrq.gov/nisoverview.jsp.

### 2.2 Study population

We identified adult hospitalizations (≥18 years) between 2017 and 2022 from the NIS. We identified patients with hematologic malignancies using International Classification of Diseases, Tenth Revision (ICD-10) diagnosis codes for acute lymphoblastic leukemia (ALL), multiple myeloma (MM), and non-Hodgkin lymphoma (NHL) recorded in either the primary diagnosis field (DX1) or secondary diagnosis fields (DX2-DX40). To ensure mutually exclusive disease categories, we excluded hospitalizations with >1 hematologic malignancy code across diagnosis fields, as well as admissions with overlapping hematologic malignancy codes appearing simultaneously in both primary and secondary diagnosis positions. Among the remaining hospitalizations, we identified patients who underwent CAR T-cell therapy during the same admission using ICD-10 procedure codes recorded in the procedure fields (PR1-PR25). We included only admissions with a single qualifying hematologic malignancy diagnosis and a documented CAR T-cell procedure in the final analytical cohort and subsequently categorized the cohort into patients with and without disseminated intravascular coagulation (DIC).

We identified ALL using ICD-10 diagnosis code C91.0, MM using code C90, and NHL using codes C82, C83, C84, C85, C86, and C88. We identified CAR T-cell therapy administration using the following ICD-10 procedure codes: XW033C3, XW043C3, XW24376, XW23346, XW24346, XW23376, XW033C7, XW033G7, XW033H7, XW033J7, XW033K7, XW033M7, XW033N7, XW043C7, XW043G7, XW043H7, XW043J7, XW043K7, XW043M7, XW043N7, XW033A7, and XW043A7. We identified DIC using ICD-10 diagnosis code D65. Where available, we cited published validation studies reporting positive predictive value (PPV), sensitivity, and specificity for these ICD-10 codes. Validation metrics for each outcome definition are summarized in Supplementary Table 9.

### 2.3 Patient and hospitalization characteristics

Our study variables encompassed patient demographics (age, sex, and race/ethnicity), insurance, and socioeconomic characteristics (insurance type/primary payer: Medicare, Medicaid, private insurance, or self-pay; and median household income quartile based on the patient’s ZIP code), and hospital teaching status/location (teaching vs nonteaching). Clinical variables included medical comorbidities, as well as both primary and secondary outcomes. We used the Charlson Comorbidity Index (CCI) to assess and stratify patients according to their comorbidity burden. As no patients had a CCI score of 0 or 1, CCI was categorized as 2 and ≥3. The CCI is a validated and widely used tool for estimating 1-year mortality risk by assigning weighted scores to specific comorbid conditions based on their prognostic impact. These conditions include myocardial infarction, congestive heart failure, peripheral vascular disease, cerebrovascular disease, dementia, chronic pulmonary disease, connective tissue disease, peptic ulcer disease, mild and moderate-to-severe liver disease, diabetes mellitus (with and without chronic complications), hemiplegia or paraplegia, moderate-to-severe renal disease, solid malignancies, leukemia, lymphoma, metastatic solid tumors, and HIV/AIDS. The cumulative score reflects the overall burden of comorbidity, with higher scores corresponding to increased risk of mortality and poorer clinical outcomes. In addition, we identified and included medical comorbidities such as sepsis, CRS, SARS-CoV-2 infection, hypertension, dyslipidemia, and atrial fibrillation, and treatments such as long-term antiplatelet and anticoagulant use based on ICD-10 codes, as detailed in Supplementary Table 1.

### 2.4 Outcomes measured

Our primary outcome was all-cause in-hospital mortality among patients with ALL, MM, and NHL who received CAR T-cell therapy, comparing those with DIC with those without DIC. Secondary outcomes were categorized into 3 principal domains. The first domain, organ dysfunction and critical care utilization, encompassed acute kidney injury (AKI), renal replacement therapy (RRT), respiratory failure (RF), mechanical ventilation use (MV), all-cause shock (shock), and vasopressor use. The second domain, thrombotic and hemorrhagic complications and interventions included acute venous thromboembolism (VTE)—comprising deep vein thrombosis and pulmonary embolism—acute coronary syndrome (ACS), gastrointestinal hemorrhage (GIH), nontraumatic intracranial hemorrhage (ICH), and transfusion requirements, such as red blood cell transfusion (RBC), platelet transfusion, and cryoprecipitate and fresh frozen plasma (FFP). The third domain, overall health care resource utilization, was assessed using length of hospital stay (LOS) and total hospitalization charges (TOTCHG).

### 2.5 Statistical analysis

All analyses accounted for the complex survey design of the NIS, including stratification, clustering, and weighting, using appropriate survey procedures. We compared continuous variables using independent Student’s *t*-test and categorical variables using Pearson chi-squared test. For outcome analyses, we applied logistic regression for binary outcomes and linear regression for continuous outcomes. To account for potential confounders, we performed multivariable regression analyses. Multivariable regression models were adjusted for the following patient-level confounders: age, sex, race/ethnicity, CCI, patient zip code income quartile, primary payer/insurance type, and comorbid conditions including sepsis, CRS, SARS-CoV-2, dyslipidemia, hypertension, atrial fibrillation, and long-term anticoagulant or antiplatelet use. These models were also adjusted for the hospital-level confounder, specifically, and hospital teaching status/location (rural, urban nonteaching, and urban teaching). We excluded patients with missing data for any of the following variables: age, sex, race/ethnicity, CCI, patient zip code income quartile, primary payer/insurance type, and hospital teaching status/location. Moreover, some covariate categories were automatically omitted in outcome-specific regression models due to sparse observations or perfect prediction. Details of omitted covariate categories are provided in Supplementary Tables 2 to 7. CAR T-cell therapy hospitalizations were predominantly concentrated in urban teaching hospitals, resulting in limited variability in hospital teaching status/location, which was not retained in all models. The CCI was presented categorically for descriptive analyses but modeled as an ordinal variable in regression analyses to preserve information and improve statistical efficiency. Additionally, we constructed a second logistic regression model that included only variables associated with the outcome of interest in univariable analysis at a significance level of *P* < .20. Unless otherwise specified, primary results are reported from the fully adjusted multivariable regression models. We conducted all statistical analyses using STATA version 18.0 Basic Edition (StataCorp) and considered 2-tailed *P* values of <.05 to be statistically significant.

## 3 Results

We identified 10,450 weighted adult hospitalizations with NHL, MM, or ALL who received CAR T-cell therapy between 2017 and 2022. Of these, 7620 (72.9%) had NHL, 2075 (19.9%) had MM, and 755 (7.2%) had ALL. DIC was diagnosed in 220 hospitalizations (2.1%), while 10,230 (97.9%) did not have DIC. Among patients who developed DIC, 135 (61.4%) had NHL, 50 (22.7%) had MM, and 35 (15.9%) had ALL. Compared with those without DIC, patients with DIC had a significantly higher proportion in the lowest income quartile (29.6% vs 17.3%; *P* = .038), self-pay status (6.8% vs 0.8%, *P* < .001), and sepsis (4.6% vs 0.8%; *P* = .007), along with a lower proportion of Hispanic patients (2.3% vs 12.2%; *P* = .046) (Table 1).

**Table 1:** Patient and hospital characteristics.

Characteristic	Patients with DIC	Patients without DIC	*P*
Patient characteristics			
No. (%) of patients	220 (2.1)	10,230 (97.9)	—
Mean age (y)	57.6	60.3	.209
Female	75 (34.1)	3920 (38.3)	.615
Race/ethnicity			
White	165 (75.0)	7,415 (72.5)	.722
Black	20 (9.1)	725 (7.1)	.618
Hispanic	5 (2.3)	1250 (12.2)	.046
Asian or Pacific Islander	15 (6.8)	415 (4.1)	.460
Native American	5 (2.3)	45 (0.4)	.093
Other	10 (4.6)	380 (3.7)	.775
Charlson Comorbidity index score			
2	25 (11.4)	1260 (12.3)	.839
≥3	195 (88.6)	8970 (87.7)	.839
Patient’s zip code income quartile			
First	65 (29.6)	1765 (17.3)	.038
Second	60 (27.3)	2200 (21.5)	.384
Third	40 (18.2)	2740 (26.8)	.195
Fourth	55 (25.0)	3525 (34.5)	.178
Insurance type			
Medicare	70 (31.8)	4265 (41.7)	.142
Medicaid	20 (9.1)	750 (7.3)	.653
Private	115 (52.3)	5130 (50.2)	.761
Self-pay	15 (6.8)	85 (0.8)	<.001
Hospital teaching status/location			
Rural	0 (0.0)	20 (0.2)	.830
Urban nonteaching	0 (0.0)	35 (0.3)	.876
Urban teaching	220 (100.0)	10,175 (99.5)	.815
Comorbidities			
Sepsis	10 (4.6)	80 (0.8)	.007
Cytokine release syndrome	90 (40.9)	4130 (40.4)	.945
SARS-CoV-2	5 (2.3)	130 (1.3)	.516
Dyslipidemia	55 (25.0)	2565 (25.1)	.992
Hypertension	80 (36.4)	4495 (43.9)	.337
Atrial fibrillation	35 (15.9)	1220 (11.9)	.432
Long-term anticoagulation use	10 (4.6)	1050 (10.3)	.220
Long-term antiplatelet use	10 (4.6)	731 (7.1)	.516

Values are given as *n* (%).

DIC, disseminated intravascular coagulation.

### 3.1 In-hospital mortality based on DIC status

Among patients who developed DIC, the in-hospital mortality rate was 22.7%, markedly higher than patients who did not develop DIC, which was 2.4% (Table 2). In both univariable and multivariable analyses, adjusting for patient- and hospital-level confounders, DIC was independently associated with increased in-hospital mortality (adjusted odds ratio [aOR], 10.96; 95% confidence interval [CI], 4.78-25.09; *P* < .001) (Table 3). These findings remained robust in a secondary model adjusted only for variables associated with increased mortality at a univariable significance threshold of *P* < .20, including race (Asian or Pacific Islander), CCI, dyslipidemia, atrial fibrillation, and long-term anticoagulation use (aOR, 10.74; 95% CI, 4.88-23.64; *P* < .001) (Supplementary Table 4).

**Table 2:** Rates of mortality and other in-hospital outcomes.

Outcome	Patients with DIC	Patients without DIC
Primary outcome		
Died	50 (22.7)	245 (2.4)
Secondary outcomes		
AKI	105 (47.7)	1410 (13.8)
RF	85 (38.6)	600 (5.9)
Shock	95 (43.2)	375 (3.7)
RRT	25 (11.4)	135 (1.3)
MV	90 (40.9)	315 (3.1)
Vasopressor	50 (22.7)	220 (2.2)
VTE	15 (6.8)	255 (2.5)
ACS	5 (2.3)	50 (0.5)
GIH	35 (15.9)	135 (1.3)
ICH	10 (4.6)	120 (1.2)
RBC	50 (22.7)	980 (9.6)
Platelet	45 (20.5)	460 (4.5)
Cryoprecipitate/FFP	40 (18.2)	190 (1.9)

ACS, acute coronary syndrome; AKI, acute kidney injury; DIC, disseminated intravascular coagulation; FFP, fresh frozen plasma; GIH, gastrointestinal hemorrhage; ICH, intracranial hemorrhage; MV, mechanical ventilation; RBC, red blood cell; RF, respiratory failure; RRT, renal replacement therapy; VTE, venous thromboembolism.

**Table 3:** CAR T-cell therapy outcomes in patients with DIC: crude and adjusted ORs.

Outcome	Crude OR (95% CI)	Adjusted OR (95% CI)
Died	11.99 (5.91-24.30)	10.96 (4.78-25.09)
AKI	5.71 (3.12-10.44)	6.01 (3.05-11.82)
RF	10.11 (5.24-19.48)	9.96 (4.90-20.26)
Shock	19.97 (10.00-39.89)	18.55 (8.60-40.01)
RRT	9.59 (3.68-24.99)	10.63 (3.30-34.23)
MV	21.79 (11.28-42.10)	22.94 (11.21-46.93)
Vasopressor	13.38 (6.13-29.19)	13.52 (5.55-32.92)
VTE	2.86 (0.87-9.42)	2.94 (0.82-10.52)
ACS	4.73 (0.53-42.16)	4.39 (0.24-82.00)
GIH	14.15 (5.82-34.39)	12.41 (4.36-35.35)
ICH	4.01 (0.93-17.33)	3.88 (0.74-20.20)
RBC	2.78 (1.28-6.02)	2.86 (1.30-6.29)
Platelet	5.46 (2.39-12.46)	5.67 (2.32-13.86)
Cryoprecipitate/FFP	11.74 (4.65-29.66)	12.70 (4.48-35.98)

Multivariable regression models were adjusted for the following covariates: age, sex, race/ethnicity, CCI, patient zip code income quartile, primary payer/insurance type, hospital teaching status/location, and comorbid conditions including sepsis, cytokine release syndrome, severe acute respiratory syndrome coronavirus 2, dyslipidemia, hypertension, atrial fibrillation, and long-term anticoagulant or antiplatelet use.

ACS, acute coronary syndrome; AKI, acute kidney injury; CAR, chimeric antigen receptor; CI, confidence interval; DIC, disseminated intravascular coagulation; FFP, fresh frozen plasma; GIH, gastrointestinal hemorrhage; ICH, intracranial hemorrhage; MV, mechanical ventilation; OR, odds ratio; RBC, red blood cell; RF, respiratory failure; RRT, renal replacement therapy; VTE, venous thromboembolism.

### 3.2 Organ dysfunction and critical care support

Among patients who developed DIC, the rates of AKI (47.7% vs 13.8%), RF (38.6% vs 5.9%), and shock (43.2% vs 3.7%) were significantly higher (Table 2). In both univariable and multivariable analyses, adjusting for patient- and hospital-level confounders, DIC was independently associated with the development of AKI (aOR, 6.01; 95% CI, 3.05-11.82; *P* < .001), RF (aOR, 9.96; 95% CI, 4.90-20.26; *P* < .001), and shock (aOR, 18.55; 95% CI, 8.60-40.01; *P* < .001) (Table 3).

Furthermore, patients with DIC demonstrated significantly higher rates of critical care interventions, including RRT (11.4% vs 1.3%), MV (40.9% vs 3.1%), and vasopressor use (22.7% vs 2.2%) (Table 2). In both univariable and multivariable models, DIC remained independently associated with increased odds of RRT (aOR, 10.63; 95% CI, 3.30-34.23; *P* < .001), MV (aOR, 22.94; 95% CI, 11.21-46.93; *P* < .001), and vasopressor (aOR, 13.52; 95% CI, 5.55-32.92; *P* < .001) utilization (Table 3).

### 3.3 Thrombosis, hemorrhage, and blood product utilization

Patients with DIC experienced a higher rate of thromboembolic complications than their counterparts without DIC. Specifically, the rate of VTE was elevated (6.8% vs 2.5%), as was the rate of ACS (2.3% vs 0.5%) (Table 2). However, after adjusting for patient- and hospital-level confounders, DIC was not significantly associated with either VTE (aOR, 2.94; 95% CI, 0.82-10.52; *P* = .097) or ACS (aOR, 4.39; 95% CI, 0.24-82.0; *P* = .321) (Table 3).

In addition, patients with DIC exhibited significantly higher rates of GIH (15.9% vs 1.3%) and ICH (4.6% vs 1.2%) than those without DIC (Table 2). However, after adjusting for potential confounders, DIC only remained significantly associated with increased odds of GIH (aOR, 12.41; 95% CI, 4.36-35.35; *P* < .001). The association between DIC and ICH did not reach statistical significance (aOR, 3.88; 95% CI, 0.74-20.20; *P* = .108) (Table 3).

DIC was also associated with significantly higher rates of blood product transfusion. Specifically, transfusion rates were higher for RBC (22.7% vs 9.6%), platelets (20.5% vs 4.5%), and cryoprecipitate/FFP (18.2% vs 1.9%) transfusions (Table 2). After adjusting for patient- and hospital-level confounders, DIC remained independently associated with increased need for RBC (aOR, 2.86; 95% CI, 1.30-6.29; *P* = .009), platelet (aOR, 5.67; 95% CI, 2.32-13.86; *P* < .001), and cryoprecipitate/FFP (aOR, 12.70; 95% CI, 4.48-35.98; *P* < .001) transfusions (Table 3).

### 3.4 Hospital length of stay and total hospitalization charges based on DIC status

The crude mean LOS and TOTCHG for CAR T-cell therapy recipients with and without DIC are presented in Table 4. After adjustment for patient- and hospital-level confounders, DIC was independently associated with increased LOS (β = +17.6 days; 95% CI, 10.16-25.08; *P* < .001) and higher TOTCHG (β = +$523,323; 95% CI, $82,053-$964,593; *P* = .020).

**Table 4:** Crude mean LOS and TOTCHG.

Outcome	Patients with DIC	Patients without DIC
Crude mean LOS (d)	36.0 (28.6–43.3)	17.1 (16.4–17.9)
Crude mean TOTCHG ($)	1,747,822 (1,302,865-2,192,780)	1,235,012 (1,090,737-1,379,287)

DIC, disseminated intravascular coagulation; LOS, length of stay; TOTCHG, total hospitalization charges.

## 4 Discussion

In this large, nationally representative cohort, we observed DIC among 2.1% of patients undergoing CAR T-cell therapy. Previously reported rates of 7% to 28% were observed in smaller single-center studies [10]. Importantly, we identified significant demographic disparities, with patients in the lowest income quartile and those with self-pay status disproportionately represented in the DIC cohort compared with the non-DIC cohort. These findings highlight the need for equity-focused strategies to identify at-risk populations and implement targeted monitoring.

DIC was associated with markedly worse inpatient outcomes. Patients with DIC had significantly higher in-hospital mortality (22.7% vs 2.4%), and DIC was strongly associated with increased odds of mortality (aOR, 10.96). This is consistent with prior adverse event registry reports, which documented fatality rates as high as 59.6% for DIC following CAR T-cell therapy, with higher mortality observed after axicabtagene ciloleucel than after tisagenlecleucel (66.7% vs 57.3%) [9]. These findings collectively emphasize DIC as a severe and life-threatening complication of CAR T-cell therapy, reinforcing the critical need for early recognition and aggressive intervention.

Organ dysfunction and increased need for intensive care were also disproportionately observed among CAR T-cell therapy patients who developed DIC. In this group, the odds of AKI, RF, and shock were approximately 6-fold, 10-fold, and 19-fold higher, respectively. Although causality cannot be established, these findings demonstrate a strong association between DIC and organ dysfunction in this population. A study by Helms et al. [11] in patients with septic shock found that DIC was independently associated with more severe forms of AKI, even after adjusting for illness severity and other clinical factors. This included stage 3 AKI, as defined by the Kidney Disease: Improving Global Outcomes classification, as well as cases requiring RRT. Consistent with these results, our study identified ∼10-fold higher odds of RRT use among patients with DIC. They also reported that higher International Society on Thrombosis and Hemostasis (ISTH) DIC scores correlated with a greater proportion of patients with stage 3 AKI. Further supporting this link, a study by Xu et al. [12] in patients with septic shock from intra-abdominal infection found that coagulation biomarkers such as activated partial thromboplastin time, prothrombin time, and D-dimer levels measured at intensive care unit (ICU) admission were independently associated with AKI. Although, to our knowledge, no prior studies have directly linked DIC with AKI in patients receiving CAR T-cell therapy, these findings, along with those of the present study, support a potential connection. The pathophysiological features of DIC, including microvascular thrombosis and resulting organ ischemia, may help explain this association and merit further investigation.

RF is a well-recognized complication of CAR T-cell therapy [13,14]. In our study, patients who developed DIC not only faced a higher risk of RF but also demonstrated a significantly increased need for MV, with a 23-fold rise compared with those without DIC. While a definitive causal relationship cannot be established, the data reveal a strong association between DIC and RF in this patient population. Prior work in acute respiratory distress syndrome populations has shown that DIC is closely linked to multiorgan dysfunction syndrome (MODS). In this multicenter cohort, DIC independently predicted the development of MODS, with higher mortality observed among those with persistent DIC [15]. In a multicenter ICU cohort of obstetrical patients, DIC was strongly associated with organ dysfunction, with DIC patients experiencing significantly higher rates of MODS/death and increased organ failure, including respiratory, renal, and neurologic compromise, compared with those without DIC [16]. These findings underscore DIC as a critical driver of multiorgan failure across diverse clinical settings and support our observation that CAR T-cell therapy recipients who develop DIC experience disproportionately higher rates of RF and AKI, consistent with the broader pathophysiological relationship between coagulopathy and multiorgan dysfunction.

Our findings highlight that DIC was associated with significantly higher rates of thrombotic and hemorrhagic complications, as well as increased blood product utilization. This is consistent with the pathophysiology of DIC, characterized by widespread coagulation activation, consumption of clotting factors, and secondary fibrinolysis. Although VTE and ACS were more frequent in patients with DIC, these associations were not statistically significant after adjustment, echoing prior observations that clinical thrombosis does not always mirror laboratory evidence of procoagulant activity in malignancy-associated DIC [17,18]. Johnsrud et al. [8] similarly attributed post–CAR T-cell thrombotic events more to underlying malignancy or hospitalization than to DIC itself.

Hemorrhagic complications, however, showed stronger associations. GIH had a significant aOR of 12.41 in the DIC group, aligning with reports of mucosal bleeding in consumptive coagulopathy [5]. While ICH occurred more often in the DIC group, statistical significance was not achieved, likely due to limited power, paralleling findings in the (a phase 1, first-in-human study of LCAR-B38M CAR-T cells in patients with relapsed/refractory multiple myeloma) LEGEND-2 trial [6]. Additional studies have reported increased bleeding risk with high-grade CRS and hemophagocytic syndromes [1,9].

Transfusion needs were also markedly elevated in patients with DIC, with significant associations for red blood cell, platelet, and plasma transfusions even after adjustment. This mirrors findings from the study by Mei et al. [7], who reported high transfusion demand in CAR T-cell–treated patients with coagulopathy, and from that of Xia et al. [2], who identified transfusion burden as a major resource utilization driver [2]. Variable transfusion thresholds across institutions may influence both outcomes and costs. A Cochrane review by Radford et al. [19] found that restrictive transfusion strategies in hematologic malignancies reduced product use without compromising survival, although CAR T–specific data remain limited. The DESCAR-T registry showed that over half of patients with large B-cell lymphoma required transfusions postinfusion, with early transfusion associated with poorer survival and reflective of higher toxicity burden [20].

The pathogenesis of DIC in this context likely involves cytokine-driven inflammation, endothelial injury, and tissue factor expression. Song et al. [9] identified DIC as a fatal complication linked to severe CRS and macrophage activation syndrome, both associated with endothelial disruption. Dholaria et al. [21] outlined a model where cytokine-induced tissue factor expression and platelet activation promote consumptive coagulopathy. Translational studies have reinforced the role of endothelial dysfunction in these processes. Elevated angiopoietin-2 and von Willebrand factor levels correlate with CRS severity, DIC risk, and adverse outcomes. Galli et al. [22] prospectively linked angiopoietin-2 to both CRS and coagulation dysfunction, while Gavriilaki et al. [23] emphasized immune-mediated endothelial injury as a central mechanism.

In this nationally representative cohort, DIC was independently associated with increased LOS and TOTCHG. These findings are consistent with prior studies linking DIC to adverse outcomes in hematologic malignancies. Franchini et al. [24] described DIC as both a marker and mediator of complications, including hemorrhage, organ dysfunction, and delayed recovery. Similar associations have been reported in stem cell transplantation and intensive chemotherapy settings [25,26]. In the context of CAR T-cell therapy, DIC may reflect downstream effects of severe immune-mediated toxicities. Neelapu et al. [27] and Strati et al. [28] identified CRS, immune effector cell–associated neurotoxicity syndrome, and secondary hemophagocytic lymphohistiocytosis/macrophage activation syndrome as major complications associated with prolonged hospitalization [27,28]. DIC may co-occur with or follow these syndromes, driven by cytokine excess and endothelial injury. Consistent with this, Song et al. [9] demonstrated that hemophagocytic lymphohistiocytosis and DIC following CAR T-cell therapy were associated with increased ICU utilization, mortality, and LOS [9], while Johnsrud et al. [8] linked bleeding and thrombotic complications to ICU transfer and extended monitoring. The observed increase in hospital charges in patients with DIC aligns with prior studies identifying ICU-level toxicities and care intensity as major drivers of cost [29,30]. Institutional analyses further support that complications such as DIC substantially increase health care utilization [25,26], underscoring the importance of toxicity-adapted care pathways to mitigate complications and optimize resource use [31].

### 4.1 Study limitations

This study used data from the NIS, a nationally representative database that captures diagnoses and procedures using ICD-10 coding. While the NIS enables large-scale epidemiologic analyses, reliance on administrative codes introduces the potential for misclassification bias, as coded diagnoses may not fully or accurately reflect underlying clinical conditions, in general, resulting in possible overrepresentation or underrepresentation of certain diagnoses. Although prior validation studies suggest that several of our ICD-10–based outcome definitions demonstrate moderate to high specificity and PPV, sensitivity is variable, and true events may be missed. For instance, our identification of DIC was based on a strategy that does not fully capture the clinical or laboratory parameters required for definitive case ascertainment. This absence of laboratory values limits the ability to apply established DIC scoring systems. Validation studies have demonstrated that ICD-based identification of DIC yields only moderate PPV, despite maintaining high specificity, indicating that coded cases are generally accurate but that a substantial proportion of true DIC events remain uncaptured [32]. Therefore, the incidence observed in this analysis likely underestimates the actual burden of DIC among CAR T-cell recipients.

Another important limitation is the inability to distinguish individual CAR T-cell products or antigen targets using administrative data. CAR T-cell exposure was identified through ICD-10 procedure coding system procedure codes, which prior to October 2021 did not reliably differentiate among Food and Drug Administration–approved CAR T-cell therapies. For example, procedure codes such as XW033C3 and XW043C3 were used for multiple products, including tisagenlecleucel (Kymriah), axicabtagene ciloleucel (Yescarta), and idecabtagene vicleucel (Abecma). Consequently, stratified analyses by CAR T-cell target (eg, CD19 vs B-cell maturation antigen) or by individual product were not feasible. This distinction is clinically relevant because CAR T-cell constructs differ in biologic targets, underlying disease populations, treatment indications, and toxicity profiles, factors that may influence the risk and outcomes of DIC. Accordingly, these findings should be interpreted within the context of this coding limitation.

Moreover, the NIS is an administrative, discharge-level database that does not capture the timing or sequence of diagnoses and procedures within a hospitalization. ICD-10-clinical modification and ICD-10 procedure coding system codes lack intrahospitalization time stamps, preventing determination of the onset of DIC relative to CAR T-cell infusion, its temporal relationship with CRS, or its duration. Therefore, our findings reflect hospitalization-level associations rather than detailed clinical trajectories.

Additionally, although the multivariable models were adjusted for known confounders, the presence of residual or unmeasured confounding cannot be entirely excluded. The retrospective nature of the study further limits causal inference. Nevertheless, the inclusion of 6 years of data from the largest all-payer hospitalization database in the United States allowed for the aggregation of a substantial cohort of patients receiving CAR T-cell therapy, facilitating the investigation of DIC in this population, a task that would be challenging to achieve through prospective data collection due to the relative rarity of this complication.

Our analysis was restricted to complete cases, with exclusion of patients with missing data in key variables, including age, sex, race/ethnicity, CCI, median household income quartile based on patient ZIP code, primary payer/insurance type, and hospital teaching status/location. Although complete case analysis is widely used, it may introduce selection bias if data are not missing completely at random, thereby potentially limiting the representativeness of the study cohort. Additionally, several outcomes involved sparse event counts, which may have contributed to inflated effect estimates and wide CIs. Finally, the charge analysis did not account for expenditures related to postdischarge care, including short-term and long-term rehabilitation services, potentially leading to an underestimation of the overall health care charges associated with DIC in the context of CAR T-cell therapy.

### 4.2 Clinical implications and future direction

Our findings underscore the importance of early recognition and proactive monitoring of coagulation parameters in patients undergoing CAR T-cell therapy, particularly among high-risk socioeconomic groups. Routine surveillance strategies may enable timely diagnosis and intervention for DIC, potentially improving outcomes. Future research should prioritize prospective studies to elucidate the pathophysiology of CAR T-associated DIC, identify predictive biomarkers, and guide the development of risk-stratified monitoring protocols. Evaluating the safety and efficacy of thromboprophylaxis and transfusion strategies tailored to this unique population also represents a critical area for future investigation.
