# Supplementary material for: Disseminated intravascular coagulation in chimeric antigen receptor T-cell therapy: a 6-year nationwide analysis of clinical outcomes and health care resource utilization in patients with hematologic malignancies
Source: Res Pract Thromb Haemost. 2026 Jul 28;10(5):106886. doi: 10.1016/j.rpth.2026.106886 (PMC13499157; doi:10.1016/j.rpth.2026.106886)
Supplement: Supplementary file 1 [file mmc1.docx]

**Supplementary Table 1. International Classification of Diseases, Tenth Revision (ICD-10) codes for study variables**

| **Variable** | **Description** | **ICD-10 code(s)** | **Code Type** |
| --- | --- | --- | --- |
| **CAR T-cell therapy** | Chimeric antigen receptor T-cell therapy | XW033C3, XW043C3, XW24376, XW23346, XW24346, XW23376, XW033C7, XW033G7, XW033H7, XW033J7, XW033K7, XW033M7, XW033N7, XW043C7, XW043G7, XW043H7, XW043J7, XW043K7, XW043M7, XW043N7, XW033A7, XW043A7. | Procedure |
| **NHL** | Non-Hodgkin's lymphoma | C82–C86, C88 | Diagnosis |
| **MM** | Multiple myeloma | C90 | Diagnosis |
| **ALL** | Acute lymphoblastic leukemia | C91.0 | Diagnosis |
| **DIC** | Disseminated intravascular coagulation | D65 | Diagnosis |
| **CRS** | Cytokine Release Syndrome | D89831, D89832, D89833, D89834, D89835, D89839, T8082 | Diagnosis |
| **Sepsis** | Sepsis | A021, A227, A267, A327, A400, A401, A403, A408, A409, A4101, A4102, A411, A412, A413, A414, A4150, A4151, A4152, A4153, A4159, A4181, A4189, A419, A427, A5486, B377, T8144XA, T8144XD, T8144XS, R6520 | Diagnosis |
| **SARS-CoV-2 infection** | Severe acute respiratory syndrome coronavirus 2 infection | U071 | Diagnosis |
| **Dyslipidemia** | Dyslipidemia | E780, E784, E785, E789 | Diagnosis |
| **Hypertension** | Hypertension | I10, I11, I12, I13, I15 | Diagnosis |
| **Atrial fibrillation** | Atrial fibrillation | I480, I481, I482, I4891 | Diagnosis |
| **Long-term anticoagulant use** | Long-term anticoagulant use | Z7901 | Diagnosis |
| **Long-term antiplatelet use** | Long-term antiplatelet use | Z7902, Z7982 | Diagnosis |
| **AKI** | Acute kidney injury | N170, N171, N172, N178, N179 | Diagnosis |
| **RRT** | Renal replacement therapy | 5A1D70Z, 5A1D90Z, 5A1D80Z | Procedure |
| **RF** | Respiratory failure | J9600–J9692, R092, J80, J95821, J95822 | Diagnosis |
| **MV** | Mechanical ventilation use | 5A1945Z, 5A1955Z, 5A1935Z, 0BH17E7, 0BH18E7, 09HN7BZ, 0BH07DZ | Procedure |
| **Shock** | All-cause shock | R570, R571, R578, R579, R6521 | Diagnosis |
| **Vasopressor** | Vasopressor use | 3E030XZ, 3E033XZ, 3E040XZ, 3E043XZ, 3E050XZ, 3E053XZ, 3E060XZ, 3E063XZ | Procedure |
| **VTE** | Acute venous thromboembolism (DVT and PE) | I2601, I2609, I2692–I2699, I824 | Diagnosis |
| **ACS** | Acute coronary syndrome | I200, I201, I208, I209, I210, I2101, I2102, I2109, I211, I212, I213, I214, I219, I22, I240, I248, I249 | Diagnosis |
| **GIH** | Gastrointestinal hemorrhage | K920, K2081, K2091, K2101, K2211, K226, K250, K252, K260, K262, K270, K272, K280, K282, K2901, K2971, K2981, K2991, K31811, K3182, I8501, I8511, K762, K2961, K921, K51411, K51511, K51811, K5521, K5701, K5711, K5713, K5721, K5731, K5733, K5741, K5751, K5753, K5781, K5791, K5793, K625, K9401, K9411, K922 | Diagnosis |
| **ICH** | Non-traumatic intracranial hemorrhage | I610–I619, I629, I6000–I6052, I606–I609, I6200–I6203, I621 | Diagnosis |
| **RBC** | Red blood cell transfusion | 30230N1, 30233N1, 30233P1, 30240N1, 30243N1, 30250N1, 30260N1, 30263P1, 30253N1, 30233N0, 30243N0, 30273N1, 30277N1, 30233P0, 30243P0 | Procedure |
| **Platelet** | Platelet transfusion | 30230R1, 30233R1, 30240R1, 30243R1, 30243R0, 30273R1, 30277R1 | Procedure |
| **FFP** | Fresh frozen plasma transfusion | 30233L0, 30233L1, 30243L0, 30243L1, 30273L1, 30233K0, 30233K1, 30243K0, 30243K1, 30273K1, 30277L1, 30277K1 | Procedure |
| **Cryo** | Cryoprecipitate transfusion | 30233M0, 30233M1, 30243M0, 30243M1, 30273M1, 30277M1, 30233D1, 30243D1 | Procedure |

**Supplementary Table 2. Univariable logistic regression analyses**

| **Covariate** | **Died OR (95% CI), p-value** | **AKI OR (95% CI), p-value** | **RF OR (95% CI), p-value** | **Shock OR (95% CI), p-value** | **RRT OR (95% CI), p-value** | **MV OR (95% CI), p-value** | **Vasopressor OR (95% CI), p-value** |
| --- | --- | --- | --- | --- | --- | --- | --- |
| DIC | 11.99 (5.91–24.30), <0.001 | 5.71 (3.12–10.45), <0.001 | 10.11 (5.24–19.48), <0.001 | 19.97 (10.00–39.89), <0.001 | 9.59 (3.68–25.00), <0.001 | 21.79 (11.28–42.10), <0.001 | 13.38 (6.13–29.19), <0.001 |
| Age | 1.00 (0.98–1.02), 0.885 | 1.01 (1.00–1.02), 0.004 | 1.00 (0.99–1.01), 0.991 | 1.00 (0.99–1.01), 0.928 | 1.00 (0.98–1.03), 0.738 | 0.99 (0.98–1.00), 0.196 | 1.01 (0.99–1.03), 0.495 |
| Sex/Female | 0.89 (0.52–1.53), 0.674 | 0.51 (0.39–0.67), <0.001 | 0.70 (0.47–1.03), 0.069 | 1.00 (0.66–1.53), 0.989 | 0.53 (0.24–1.19), 0.125 | 0.71 (0.43–1.17), 0.179 | 1.11 (0.65–1.92), 0.697 |
| **Race/Ethnicity** | | | | | | | |
| Black | 0.99 (0.35–2.81), 0.989 | 1.18 (0.76–1.85), 0.464 | 0.79 (0.38–1.63), 0.517 | 0.88 (0.38–2.05), 0.767 | 1.46 (0.43–4.95), 0.540 | 1.07 (0.47–2.45), 0.866 | 0.72 (0.22–2.37), 0.590 |
| Hispanic | 0.73 (0.30–1.80), 0.496 | 0.77 (0.50–1.17), 0.213 | 0.88 (0.48–1.61), 0.681 | 0.96 (0.49–1.87), 0.907 | 0.86 (0.28–2.61), 0.792 | 0.95 (0.44–2.04), 0.899 | 0.57 (0.21–1.57), 0.277 |
| Asian or Pacific Islander | 2.22 (0.85–5.79), 0.103 | 1.24 (0.71–2.17), 0.457 | 1.04 (0.44–2.48), 0.930 | 0.76 (0.23–2.50), 0.649 | 2.57 (0.75–8.82), 0.132 | 0.93 (0.28–3.04), 0.898 | 1.27 (0.37–4.31), 0.703 |
| Native American | 4.00 (0.46–34.93), 0.210 | 1.46 (0.28–7.63), 0.650 | 1.54 (0.18–13.23), 0.694 | 5.24 (0.97–28.25), 0.054 | 1 (N/A), N/A | 2.84 (0.33–24.54), 0.342 | 3.90 (0.45–33.80), 0.217 |
| Other | 1.44 (0.26–7.89), 0.675 | 0.96 (0.47–1.98), 0.916 | 0.95 (0.41–2.20), 0.904 | 0.84 (0.30–2.36), 0.739 | 1.87 (0.53–6.64), 0.331 | 1.75 (0.75–4.11), 0.197 | 0.46 (0.09–2.40), 0.354 |
| CCI | 4.09 (0.98–17.14), 0.054 | 1.74 (1.09–2.78), 0.020 | 2.36 (1.11–5.02), 0.026 | 1.53 (0.78–3.02), 0.218 | 2.12 (0.30–14.96), 0.450 | 3.76 (1.28–11.06), 0.016 | 1.38 (0.59–3.27), 0.459 |
| **Insurance type** | | | | | | | |
| Medicaid | 1.23 (0.46–3.31), 0.680 | 0.74 (0.44–1.27), 0.278 | 1.30 (0.63–2.67), 0.476 | 0.80 (0.30–2.10), 0.647 | 1.13 (0.25–5.14), 0.877 | 1.83 (0.75–4.45), 0.183 | 0.70 (0.20–2.43), 0.571 |
| Private | 1.12 (0.60–2.07), 0.723 | 0.84 (0.64–1.10), 0.199 | 1.59 (1.08–2.34), 0.020 | 1.21 (0.77–1.93), 0.408 | 1.67 (0.77–3.62), 0.198 | 2.00 (1.16–3.45), 0.012 | 0.93 (0.52–1.67), 0.803 |
| Self-pay | 1 (N/A), N/A | 0.59 (0.13–2.59), 0.481 | 0.98 (0.12–7.79), 0.988 | 4.19 (0.94–18.73), 0.060 | 1 (N/A), N/A | 1 (N/A), N/A | 1 (N/A), N/A |
| Hospital teaching status/location | 1 (N/A), N/A | 0.76 (0.29–2.00), 0.580 | 0.70 (0.27–1.85), 0.471 | 0.47 (0.18–1.25), 0.128 | 1 (N/A), N/A | 1 (N/A), N/A | 1 (N/A), N/A |
| **ZIP income quartile** | | | | | | | |
| 2nd | 1.01 (0.45–2.27), 0.976 | 0.84 (0.56–1.26), 0.392 | 0.72 (0.41–1.27), 0.251 | 0.88 (0.47–1.66), 0.695 | 2.18 (0.56–8.43), 0.259 | 0.94 (0.51–1.74), 0.848 | 1.27 (0.51–3.18), 0.613 |
| 3rd | 0.60 (0.27–1.33), 0.205 | 0.69 (0.49–0.98), 0.037 | 0.60 (0.37–0.97), 0.037 | 0.61 (0.33–1.15), 0.130 | 1.99 (0.50–7.87), 0.326 | 0.80 (0.46–1.39), 0.421 | 1.33 (0.55–3.20), 0.529 |
| 4th | 0.89 (0.43–1.85), 0.758 | 0.85 (0.61–1.20), 0.355 | 0.64 (0.41–1.00), 0.053 | 0.72 (0.41–1.26), 0.247 | 2.06 (0.58–7.34), 0.263 | 0.56 (0.31–1.01), 0.053 | 0.73 (0.30–1.81), 0.502 |
| Sepsis | 2.04 (0.27–15.64), 0.492 | 6.05 (2.39–15.29), <0.001 | 4.17 (1.34–12.98), 0.014 | 6.29 (2.01–19.70), 0.002 | 3.87 (0.49–30.33), 0.197 | 3.15 (0.71–14.05), 0.132 | 2.24 (0.30–16.81), 0.432 |
| CRS | 0.94 (0.53–1.67), 0.837 | 1.06 (0.79–1.41), 0.692 | 1.20 (0.84–1.72), 0.322 | 1.15 (0.73–1.80), 0.543 | 1.15 (0.59–2.23), 0.677 | 0.91 (0.57–1.47), 0.705 | 1.38 (0.78–2.45), 0.265 |
| SARS-CoV-2 | 1 (N/A), N/A | 1.70 (0.74–3.89), 0.210 | 0.54 (0.07–4.15), 0.558 | 1 (N/A), N/A | 1 (N/A), N/A | 0.95 (0.12–7.30), 0.963 | 1 (N/A), N/A |
| Dyslipidemia | 0.60 (0.30–1.21), 0.152 | 0.98 (0.74–1.30), 0.888 | 1.03 (0.66–1.60), 0.906 | 0.85 (0.53–1.38), 0.519 | 0.83 (0.35–1.99), 0.683 | 0.79 (0.44–1.40), 0.418 | 1.51 (0.85–2.69), 0.160 |
| Hypertension | 1.01 (0.59–1.74), 0.965 | 1.71 (1.34–2.17), <0.001 | 1.46 (1.04–2.05), 0.031 | 0.95 (0.61–1.46), 0.813 | 1.90 (0.98–3.65), 0.056 | 0.93 (0.59–1.46), 0.743 | 0.88 (0.49–1.58), 0.668 |
| Atrial fibrillation | 2.13 (1.11–4.09), 0.023 | 1.56 (1.13–2.15), 0.006 | 1.80 (1.16–2.80), 0.009 | 1.93 (1.13–3.28), 0.016 | 3.43 (1.55–7.56), 0.002 | 1.86 (1.03–3.34), 0.038 | 2.66 (1.30–5.41), 0.007 |
| Long-term anticoagulant use | 0.15 (0.02–1.03), 0.053 | 0.73 (0.45–1.18), 0.204 | 0.68 (0.36–1.31), 0.249 | 0.59 (0.26–1.33), 0.205 | 0.59 (0.15–2.25), 0.436 | 0.70 (0.30–1.62), 0.405 | 0.90 (0.36–2.24), 0.824 |
| Long-term antiplatelet use | 0.45 (0.11–1.84), 0.269 | 0.86 (0.52–1.41), 0.549 | 1.16 (0.64–2.09), 0.627 | 0.57 (0.21–1.57), 0.277 | 0.42 (0.06–2.90), 0.378 | 1.05 (0.46–2.39), 0.904 | 0.50 (0.12–2.01), 0.327 |

N/A indicates estimates not calculable due to sparse data or perfect prediction

**Abbreviations:** OR, odds ratio; CI, confidence interval

**Supplementary Table 3. Univariable logistic regression analyses**

| **Covariate** | **VTE OR (95% CI), p-value** | **ACS OR (95% CI), p-value** | **GIH OR (95% CI), p-value** | **ICH OR (95% CI), p-value** | **RBC OR (95% CI), p-value** | **Platelet OR (95% CI), p-value** | **Cryo/FFP OR (95% CI), p-value** |
| --- | --- | --- | --- | --- | --- | --- | --- |
| DIC | 2.86 (0.87–9.42), 0.083 | 4.73 (0.53–42.16), 0.163 | 14.15 (5.82–34.39), <0.001 | 4.01 (0.93–17.33), 0.063 | 2.78 (1.28–6.02), 0.010 | 5.46 (2.39–12.46), <0.001 | 11.74 (4.65–29.66), <0.001 |
| Age | 1.00 (0.98–1.02), 0.624 | 1.03 (0.99–1.07), 0.105 | 0.99 (0.97–1.01), 0.428 | 0.99 (0.96–1.03), 0.749 | 1.00 (0.99–1.01), 0.408 | 1.00 (0.99–1.01), 0.961 | 1.01 (0.98–1.03), 0.651 |
| Sex/Female | 0.95 (0.55–1.63), 0.850 | 1.35 (0.47–3.86), 0.577 | 0.77 (0.37–1.59), 0.480 | 0.72 (0.31–1.66), 0.436 | 1.26 (0.93–1.69), 0.131 | 0.89 (0.58–1.37), 0.596 | 0.95 (0.55–1.67), 0.848 |
| **Race/Ethnicity** | | | | | | | |
| Black | 1.47 (0.57–3.79), 0.426 | 1.46 (0.30–7.04), 0.640 | 0.37 (0.05–2.80), 0.337 | 0.53 (0.07–3.97), 0.538 | 1.33 (0.76–2.33), 0.321 | 0.72 (0.30–1.70), 0.452 | 0.87 (0.28–2.75), 0.812 |
| Hispanic | 1.76 (0.80–3.85), 0.160 | 1.73 (0.35–8.69), 0.505 | 0.67 (0.22–2.03), 0.476 | 1.28 (0.42–3.83), 0.664 | 1.41 (0.86–2.33), 0.172 | 1.50 (0.87–2.58), 0.143 | 0.86 (0.37–2.03), 0.730 |
| Asian or Pacific Islander | 0.50 (0.07–3.60), 0.489 | 1 (N/A), N/A | 0.65 (0.09–4.84), 0.673 | 0.93 (0.12–7.13), 0.942 | 1.57 (0.79–3.11), 0.197 | 1.55 (0.65–3.70), 0.324 | 0.50 (0.07–3.47), 0.481 |
| Native American | 4.70 (0.89–24.90), 0.069 | 23.95 (2.44–235.24), 0.006 | 1 (N/A), N/A | 1 (N/A), N/A | 1 (N/A), N/A | 1 (N/A), N/A | 1 (N/A), N/A |
| Other | 1.11 (0.25–5.00), 0.888 | 1 (N/A), N/A | 1.45 (0.43–4.91), 0.549 | 1.02 (0.19–5.54), 0.979 | 0.25 (0.06–1.05), 0.059 | 0.83 (0.19–3.68), 0.802 | 1.11 (0.26–4.83), 0.886 |
| CCI | 0.94 (0.44–2.03), 0.874 | 1 (N/A), N/A | 4.69 (0.68–32.26), 0.116 | 1 (N/A), N/A | 1.13 (0.64–1.98), 0.674 | 1.46 (0.76–2.81), 0.262 | 1.48 (0.43–5.17), 0.536 |
| **Insurance type** | | | | | | | |
| Medicaid | 1.26 (0.41–3.81), 0.685 | 1 (N/A), N/A | 1.75 (0.58–5.33), 0.323 | 1.90 (0.60–6.02), 0.275 | 1.08 (0.65–1.79), 0.764 | 1.51 (0.79–2.87), 0.208 | 1.13 (0.38–3.36), 0.827 |
| Private | 1.48 (0.80–2.74), 0.208 | 0.69 (0.23–2.01), 0.494 | 1.08 (0.52–2.27), 0.835 | 0.69 (0.30–1.59), 0.381 | 1.00 (0.74–1.36), 0.980 | 0.92 (0.56–1.52), 0.750 | 0.82 (0.44–1.54), 0.544 |
| Self-pay | 1 (N/A), N/A | 1 (N/A), N/A | 1 (N/A), N/A | 1 (N/A), N/A | 2.33 (0.91–5.95), 0.077 | 1.03 (0.16–6.52), 0.972 | 4.71 (1.21–18.26), 0.025 |
| Hospital teaching status/location | 1 (N/A), N/A | 1 (N/A), N/A | 1 (N/A), N/A | 1 (N/A), N/A | 1 (N/A), N/A | 1 (N/A), N/A | 1 (N/A), N/A |
| **ZIP income quartile** | | | | | | | |
| 2nd | 1.46 (0.64–3.31), 0.371 | 3.26 (0.56–18.87), 0.187 | 1.22 (0.43–3.44), 0.709 | 1.14 (0.36–3.55), 0.827 | 1.50 (0.95–2.37), 0.081 | 1.10 (0.61–1.99), 0.751 | 1.76 (0.79–3.92), 0.166 |
| 3rd | 1.25 (0.55–2.82), 0.589 | 1.98 (0.40–9.87), 0.405 | 1.10 (0.39–3.07), 0.857 | 1.05 (0.35–3.19), 0.926 | 1.65 (1.07–2.55), 0.023 | 1.21 (0.70–2.11), 0.497 | 1.32 (0.52–3.40), 0.558 |
| 4th | 0.68 (0.28–1.66), 0.393 | 1.54 (0.16–14.90), 0.711 | 0.76 (0.29–1.98), 0.579 | 0.61 (0.19–2.00), 0.415 | 1.12 (0.67–1.88), 0.654 | 0.90 (0.45–1.79), 0.760 | 0.73 (0.31–1.72), 0.466 |
| Sepsis | 7.93 (2.18–28.88), 0.002 | 1 (N/A), N/A | 13.17 (3.61–48.10), <0.001 | 1 (N/A), N/A | 2.64 (0.87–8.08), 0.088 | 4.03 (1.14–14.29), 0.031 | 1 (N/A), N/A |
| CRS | 0.94 (0.52–1.70), 0.832 | 0.33 (0.07–1.63), 0.173 | 0.70 (0.36–1.36), 0.292 | 0.65 (0.29–1.49), 0.311 | 1.19 (0.79–1.78), 0.403 | 1.48 (0.88–2.47), 0.138 | 1.78 (0.92–3.46), 0.089 |
| SARS-CoV-2 | 1 (N/A), N/A | 1 (N/A), N/A | 1 (N/A), N/A | 6.80 (1.62–28.43), 0.009 | 1.15 (0.34–3.91), 0.829 | 1.59 (0.37–6.80), 0.534 | 1 (N/A), N/A |
| Dyslipidemia | 1.05 (0.58–1.91), 0.880 | 2.50 (0.88–7.16), 0.087 | 0.77 (0.33–1.81), 0.550 | 1.10 (0.47–2.61), 0.825 | 0.87 (0.61–1.25), 0.453 | 0.93 (0.58–1.48), 0.755 | 1.46 (0.82–2.58), 0.195 |
| Hypertension | 1.11 (0.61–2.02), 0.732 | 1.54 (0.34–6.95), 0.571 | 1.01 (0.53–1.95), 0.967 | 0.80 (0.36–1.78), 0.585 | 1.13 (0.86–1.50), 0.369 | 1.33 (0.85–2.07), 0.209 | 1.41 (0.68–2.91), 0.351 |
| Atrial fibrillation | 1.28 (0.61–2.69), 0.509 | 1.63 (0.48–5.61), 0.436 | 2.30 (1.05–5.00), 0.037 | 0.96 (0.28–3.22), 0.941 | 1.01 (0.67–1.53), 0.950 | 1.63 (0.95–2.81), 0.075 | 1.10 (0.47–2.60), 0.826 |
| Long-term anticoagulant use | 2.62 (1.27–5.43), 0.009 | 0.89 (0.11–7.31), 0.910 | 0.55 (0.13–2.33), 0.416 | 0.35 (0.05–2.60), 0.305 | 0.89 (0.54–1.47), 0.650 | 0.86 (0.42–1.75), 0.678 | 0.61 (0.18–2.03), 0.423 |
| Long-term antiplatelet use | 0.77 (0.24–2.41), 0.649 | 1.31 (0.24–7.18), 0.752 | 1 (N/A), N/A | 0.52 (0.07–3.92), 0.527 | 1.03 (0.60–1.77), 0.904 | 0.98 (0.46–2.07), 0.950 | 0.91 (0.29–2.90), 0.878 |

N/A indicates estimates not calculable due to sparse data or perfect prediction

**Abbreviations:** OR, odds ratio; CI, confidence interval

**Supplementary Table 4. Multivariable logistic regression analyses adjusted for covariates with univariable p < 0.20**

| **Covariate** | **Died OR (95% CI), p-value** | **AKI OR (95% CI), p-value** | **RRT OR (95% CI), p-value** | **RF OR (95% CI), p-value** | **MV OR (95% CI), p-value** | **Shock OR (95% CI), p-value** | **Vasopressor OR (95% CI), p-value** |
| --- | --- | --- | --- | --- | --- | --- | --- |
| DIC | 10.74 (4.88–23.64), <0.001 | 6.12 (3.15–11.91), <0.001 | 9.55 (3.12–29.20), <0.001 | 9.70 (4.85–19.42), <0.001 | 22.69 (11.15–46.18), <0.001 | 18.28 (8.55–39.08), <0.001 | 13.34 (5.92–30.07), <0.001 |
| Age | – | 1.01 (1.00–1.02), 0.084 | – | – | 1.00 (0.98–1.02), 0.792 | – | – |
| Sex/Female | – | 0.51 (0.38–0.67), <0.001 | 0.65 (0.29–1.43), 0.284 | 0.77 (0.52–1.14), 0.191 | 0.77 (0.47–1.28), 0.314 | – | – |
| **Race/Ethnicity** | | | | | | | |
| Black | 0.94 (0.33–2.66), 0.914 | – | 1.37 (0.37–4.99), 0.636 | – | 0.94 (0.38–2.32), 0.891 | 0.89 (0.35–2.23), 0.796 | – |
| Hispanic | 0.86 (0.34–2.14), 0.744 | – | 1.22 (0.36–4.10), 0.750 | – | 1.10 (0.48–2.51), 0.819 | 1.28 (0.62–2.63), 0.505 | – |
| Asian or Pacific Islander | 2.24 (0.83–6.07), 0.112 | – | 2.83 (0.72–11.04), 0.135 | – | 0.97 (0.24–3.92), 0.969 | 0.65 (0.13–3.23), 0.600 | – |
| Native American | 2.52 (0.16–39.48), 0.511 | – | 1 (N/A), N/A | – | 1.25 (0.04–38.37), 0.897 | 3.76 (0.61–23.14), 0.153 | – |
| Other | 1.27 (0.24–6.78), 0.779 | – | 1.74 (0.47–6.48), 0.411 | – | 1.57 (0.61–4.06), 0.354 | 0.79 (0.27–2.30), 0.665 | – |
| CCI | 4.46 (1.07–18.54), 0.040 | 1.59 (1.00–2.53), 0.048 | – | 2.15 (0.99–4.69), 0.053 | 3.76 (1.38–10.27), 0.010 | – | – |
| **Insurance type** | | | | | | | |
| Medicaid | – | 1.02 (0.57–1.85), 0.936 | 1.31 (0.23–7.30), 0.760 | 1.38 (0.64–2.97), 0.414 | 1.86 (0.62–5.61), 0.268 | 0.68 (0.22–2.14), 0.508 | – |
| Private | – | 1.00 (0.72–1.39), 0.988 | 1.96 (0.81–4.76), 0.137 | 1.77 (1.16–2.70), 0.008 | 2.27 (1.14–4.52), 0.019 | 1.28 (0.78–2.12), 0.331 | – |
| Self-pay | – | 0.59 (0.13–2.78), 0.506 | 1 (N/A), N/A | 0.73 (0.07–8.20), 0.800 | 1 (N/A) | 2.61 (0.42–16.25), 0.304 | – |
| Hospital teaching status/location | – | – | – | – | – | 0.39 (0.14–1.09), 0.072 | – |
| **ZIP income quartile** | | | | | | | |
| 2nd | – | 0.89 (0.59–1.36), 0.594 | – | 0.80 (0.44–1.46), 0.472 | 1.13 (0.58–2.22), 0.711 | 0.98 (0.50–1.91), 0.948 | – |
| 3rd | – | 0.77 (0.53–1.10), 0.153 | – | 0.70 (0.41–1.18), 0.183 | 1.04 (0.54–2.00), 0.906 | 0.75 (0.38–1.49), 0.409 | – |
| 4th | – | 0.93 (0.65–1.34), 0.705 | – | 0.74 (0.46–1.20), 0.223 | 0.66 (0.34–1.28), 0.219 | 0.86 (0.44–1.68), 0.667 | – |
| Sepsis | – | 7.17 (2.49–20.67), <0.001 | 3.09 (0.16–61.07), 0.459 | 3.91 (1.07–14.31), 0.040 | 2.14 (0.22–21.09), 0.515 | 5.48 (1.44–20.78), 0.012 | – |
| CRS | – | – | – | – | – | – | – |
| SARS-CoV-2 | – | – | – | – | – | – | – |
| Dyslipidemia | 0.56 (0.28–1.11), 0.098 | – | – | – | – | – | 1.43 (0.79–2.61), 0.239 |
| Hypertension | – | 1.61 (1.23–2.10), <0.001 | 1.83 (0.91–3.68), 0.092 | 1.49 (1.03–2.16), 0.035 | – | – | – |
| Atrial fibrillation | 2.66 (1.35–5.27), 0.005 | 1.16 (0.82–1.65), 0.404 | 3.47 (1.46–8.28), 0.005 | 1.77 (1.09–2.85), 0.020 | 2.04 (1.08–3.87), 0.028 | 1.99 (1.09–3.67), 0.026 | 2.52 (1.20–5.30), 0.015 |
| Long-term anticoagulant use | 0.14 (0.02–0.88), 0.036 | – | – | – | – | – | – |
| Long-term antiplatelet use | – | – | – | – | – | – | – |

N/A indicates estimates not calculable due to sparse data or perfect prediction

**Abbreviations:** aOR, adjusted odds ratio; CI, confidence interval

- indicates variable not included in the multivariable model

**Supplementary Table 5. Multivariable logistic regression analyses adjusted for covariates with univariable p < 0.20**

| **Covariate** | **VTE OR (95% CI), p-value** | **ACS OR (95% CI), p-value** | **GIH OR (95% CI), p-value** | **ICH OR (95% CI), p-value** | **RBC OR (95% CI), p-value** | **Platelet OR (95% CI), p-value** | **Cryo/FFP OR (95% CI), p-value** |
| --- | --- | --- | --- | --- | --- | --- | --- |
| DIC | 2.77 (0.72–10.63), 0.138 | 4.28 (0.28–65.56), 0.297 | 12.42 (4.69–32.89), <0.001 | 3.88 (0.84–17.82), 0.082 | 2.78 (1.26–6.14), 0.011 | 5.45 (2.24–13.24), <0.001 | 11.20 (4.21–29.83), <0.001 |
| Age | – | 1.04 (1.00–1.09), 0.039 | – | – | – | – | – |
| Sex/Female | – | – | – | – | 1.23 (0.91–1.67), 0.177 | 0.92 (0.58–1.45), 0.712 | – |
| **Race/Ethnicity** | | | | | | | |
| Black | 1.53 (0.58–4.04), 0.387 | 2.21 (0.60–8.07), 0.232 | – | – | 1.34 (0.74–2.41), 0.335 | 0.71 (0.30–1.65), 0.422 | – |
| Hispanic | 1.91 (0.87–4.17), 0.105 | 3.28 (0.59–18.33), 0.175 | – | – | 1.45 (0.85–2.47), 0.173 | 1.65 (0.94–2.90), 0.081 | – |
| Asian or Pacific Islander | 0.53 (0.07–3.86), 0.527 | 1 (N/A), N/A | – | – | 1.47 (0.71–3.04), 0.297 | 1.55 (0.63–3.78), 0.340 | – |
| Native American | 5.36 (0.86–33.21), 0.071 | 39.75 (1.40–1125.41), 0.031 | – | – | 1 (N/A), N/A | 1 (N/A), N/A | – |
| Other | 1.26 (0.28–5.70), 0.763 | 1 (N/A), N/A | – | – | 0.26 (0.06–1.07), 0.061 | 0.87 (0.19–4.03), 0.857 | – |
| CCI | – | – | 4.33 (0.69–27.12), 0.117 | – | – | – | – |
| **Insurance type** | | | | | | | |
| Medicaid | – | – | – | – | 0.93 (0.53–1.61), 0.785 | – | 1.18 (0.38-3.62), 0.776 |
| Private | – | – | – | – | 1.00 (0.73–1.38), 0.977 | – | 0.85 (0.45-1.59), 0.602 |
| Self-pay | – | – | – | – | 1.85 (0.70–4.90), 0.219 | – | 3.03 (0.97-9.46), 0.056 |
| Hospital teaching status/location | – | – | – | – | – | – | – |
| **ZIP income quartile** | | | | | | | |
| 2nd | – | 4.44 (0.64–30.90), 0.132 | – | – | 1.52 (0.95-2.42), 0.079 | – | 2.06 (0.88-4.84), 0.097 |
| 3rd | – | 2.86 (0.47–17.47), 0.255 | – | – | 1.70 (1.10-2.64), 0.018 | – | 1.62 (0.59-4.39), 0.346 |
| 4th | – | 2.41 (0.16–36.24), 0.526 | – | – | 1.16 (0.68-1.99), 0.579 | – | 0.90 (0.36-2.26), 0.826 |
| Sepsis | 7.95 (2.00–31.59), 0.003 | – | 8.95 (1.70–47.17), 0.010 | – | 2.23 (0.68–7.31), 0.187 | 3.53 (0.85–14.70), 0.083 | – |
| CRS | – | 0.30 (0.07–1.35), 0.117 | – | – | – | 1.44 (0.84–2.48), 0.181 | 1.80 (0.93–3.46), 0.080 |
| SARS-CoV-2 | – | – | – | 6.60 (1.68–25.92), 0.007 | – | – | – |
| Dyslipidemia | – | 2.33 (0.78–6.97), 0.129 | – | – | – | – | 1.50 (0.86–2.61), 0.153 |
| Hypertension | – | – | – | – | – | – | – |
| Atrial fibrillation | – | – | 2.15 (0.91–5.08), 0.081 | – | – | 1.63 (0.93–2.86), 0.087 | – |
| Long-term anticoagulant use | 2.91 (1.39–6.09), 0.005 | – | – | – | – | – | – |
| Long-term antiplatelet use | – | – | – | – | – | – | – |

N/A indicates estimates not calculable due to sparse data or perfect prediction

**Abbreviations:** aOR, adjusted odds ratio; CI, confidence interval

- indicates variable not included in the multivariable model

**Supplementary Table 6. Multivariable logistic regression analyses adjusted for all covariates included in the study**

| **Covariate** | **Died OR (95% CI), p-value** | **AKI OR (95% CI), p-value** | **RF OR (95% CI), p-value** | **Shock OR (95% CI), p-value** | **RRT OR (95% CI), p-value** | **MV OR (95% CI), p-value** | **Vasopressor OR (95% CI), p-value** |
| --- | --- | --- | --- | --- | --- | --- | --- |
| DIC | 10.96 (4.78–25.09), <0.001 | 6.01 (3.05–11.82), <0.001 | 9.96 (4.90–20.26), <0.001 | 18.55 (8.60–40.01), <0.001 | 10.63 (3.30–34.23), <0.001 | 22.94 (11.21–46.93), <0.001 | 13.52 (5.55–32.92), <0.001 |
| Age | 1.01 (0.98–1.04), 0.707 | 1.01 (1.00–1.03), 0.032 | 1.01 (0.99–1.03), 0.393 | 1.01 (0.99–1.03), 0.523 | 1.00 (0.97–1.04), 0.805 | 1.00 (0.98–1.03), 0.652 | 1.00 (0.97–1.03), 0.988 |
| Sex/Female | 0.88 (0.50–1.55), 0.664 | 0.49 (0.37–0.64), <0.001 | 0.75 (0.51–1.11), 0.151 | 1.05 (0.68–1.63), 0.824 | 0.59 (0.26–1.36), 0.219 | 0.75 (0.45–1.25), 0.269 | 1.19 (0.68–2.11), 0.539 |
| **Race/Ethnicity** | | | | | | | |
| Black | 0.90 (0.32–2.51), 0.844 | 1.18 (0.72–1.92), 0.511 | 0.61 (0.29–1.28), 0.189 | 0.86 (0.35–2.14), 0.746 | 1.63 (0.44–6.01), 0.463 | 0.92 (0.38–2.26), 0.858 | 0.77 (0.22–2.65), 0.675 |
| Hispanic | 0.86 (0.31–2.39), 0.773 | 0.98 (0.63–1.55), 0.942 | 0.98 (0.51–1.90), 0.953 | 1.26 (0.60–2.62), 0.542 | 1.26 (0.35-4.60), 0.722 | 1.06 (0.45–2.46), 0.897 | 0.68 (0.23–1.98), 0.475 |
| Asian or Pacific Islander | 2.29 (0.82–6.40), 0.115 | 1.33 (0.70–2.51), 0.384 | 0.98 (0.39–2.48), 0.968 | 0.28 (0.02–4.15), 0.357 | 2.46 (0.60-10.12),.211 | 0.94 (0.23-3.80), 0.928 | 1.59 (0.39–6.49), 0.521 |
| Native American | 2.20 (0.13–37.83), 0.587 | 1.35 (0.20–8.92), 0.757 | 0.82 (0.06–10.94), 0.879 | 3.66 (0.56–23.76), 0.174 | 1 (N/A), N/A | 1.26 (0.04–42.84), 0.899 | 2.48 (0.43–14.32), 0.310 |
| Other | 1.26 (0.24–6.61), 0.783 | 1.01 (0.49–2.05), 0.988 | 0.83 (0.34–2.02), 0.676 | 0.63 (0.07–5.57), 0.674 | 1.94 (0.54-6.96), 0.311 | 1.53 (0.60–3.90), 0.372 | 0.47 (0.08–2.85), 0.413 |
| CCI | 4.08 (0.94–17.73), 0.061 | 1.67 (1.03–2.70), 0.037 | 2.43 (1.08–5.44), 0.032 | 1.72 (0.78–3.76), 0.177 | 2.17 (0.25–18.55), 0.478 | 4.06 (1.44–11.40), 0.008 | 1.52 (0.62–3.77), 0.362 |
| **Insurance type** | | | | | | | |
| Medicaid | 1.34 (0.44–4.11), 0.606 | 1.00 (0.54–1.86), 0.993 | 1.80 (0.79–4.09), 0.160 | 0.77 (0.23–2.61), 0.674 | 1.37 (0.24–7.89), 0.724 | 1.94 (0.65–5.79), 0.236 | 0.85 (0.18–3.99), 0.835 |
| Private | 1.11 (0.50–2.50), 0.795 | 0.97 (0.70–1.36), 0.875 | 1.97 (1.20–3.22), 0.007 | 1.36 (0.75–2.45), 0.310 | 1.97 (0.74–5.25), 0.175 | 2.29 (1.15–4.57), 0.018 | 1.03 (0.45–2.36), 0.942 |
| Self-pay | 1 (N/A), N/A | 0.55 (0.11–2.65), 0.456 | 0.78 (0.06–9.99), 0.850 | 2.81 (0.51–15.49), 0.235 | 1 (N/A), N/A | 1 (N/A), N/A | 1 (N/A), N/A |
| Hospital teaching status/location | 1 (N/A), N/A | 0.56 (0.17–1.87), 0.346 | 0.59 (0.23–1.52), 0.277 | 0.39 (0.15–1.01), 0.051 | 1 (N/A), N/A | 1 (N/A), N/A | 1 (N/A), N/A |
| **ZIP income quartile** | | | | | | | |
| 2nd | 1.13 (0.48–2.62), 0.783 | 0.93 (0.60–1.44), 0.743 | 0.75 (0.41–1.38), 0.362 | 0.99 (0.50–1.97), 0.983 | 3.17 (0.81–12.35), 0.097 | 1.11 (0.56–2.18), 0.768 | 1.38 (0.48–3.95), 0.546 |
| 3rd | 0.72 (0.31–1.66), 0.442 | 0.79 (0.54–1.17), 0.243 | 0.67 (0.39–1.14), 0.139 | 0.77 (0.39–1.53), 0.457 | 2.79 (0.68–11.50), 0.156 | 1.04 (0.56–1.96), 0.894 | 1.48 (0.54–4.08), 0.447 |
| 4th | 0.93 (0.42–2.07), 0.858 | 0.95 (0.65–1.40), 0.797 | 0.68 (0.42–1.13), 0.137 | 0.86 (0.44–1.68), 0.655 | 2.55 (0.71–9.21), 0.153 | 0.63 (0.32–1.23), 0.175 | 0.74 (0.26–2.13), 0.576 |
| Sepsis | 0.85 (0.05–15.84), 0.914 | 7.43 (2.53–21.82), <0.001 | 4.06 (1.05–15.75), 0.043 | 5.18 (1.34–20.07), 0.017 | 2.42 (0.08–70.00), 0.606 | 2.04 (0.20–21.26), 0.550 | 1.43 (0.15–13.48), 0.753 |
| CRS | 1.06 (0.58–1.95), 0.841 | 1.12 (0.81–1.54), 0.491 | 1.32 (0.90–1.94), 0.160 | 1.26 (0.77–2.04), 0.360 | 1.20 (0.56–2.54), 0.640 | 1.04 (0.62–1.74), 0.882 | 1.43 (0.78–2.63), 0.251 |
| SARS-CoV-2 | 1 (N/A), N/A | 1.64 (0.63–4.25), 0.312 | 0.45 (0.05–3.89), 0.472 | 1 (N/A), N/A | 1 (N/A), N/A | 0.71 (0.05–9.27), 0.794 | 1 (N/A), N/A |
| Dyslipidemia | 0.57 (0.27–1.19), 0.133 | 0.73 (0.54–1.01), 0.056 | 0.87 (0.55–1.39), 0.561 | 0.84 (0.50–1.41), 0.509 | 0.76 (0.27–2.15), 0.608 | 0.72 (0.38–1.38), 0.319 | 1.57 (0.82–3.00), 0.71 |
| Hypertension | 1.06 (0.60–1.88), 0.849 | 1.70 (1.29–2.26), <0.001 | 1.51 (1.02–2.26), 0.041 | 1.01 (0.62–1.62), 0.983 | 1.98 (0.89–4.40), 0.095 | 0.94 (0.54–1.63), 0.825 | 0.70 (0.34–1.45), 0.340 |
| Atrial fibrillation | 2.50 (1.24–5.05), 0.011 | 1.26 (0.89–1.79), 0.189 | 1.80 (1.09–2.96), 0.021 | 2.04 (1.12–3.72), 0.020 | 3.78 (1.57–9.08), 0.003 | 2.16 (1.12–4.15), 0.021 | 2.64 (1.18–5.91), 0.018 |
| Long-term anticoagulant use | 0.13 (0.02–0.87), 0.035 | 0.62 (0.38–1.00), 0.051 | 0.60 (0.30–1.20), 0.148 | 0.60 (0.25–1.41), 0.241 | 0.41 (0.10–1.71), 0.222 | 0.67 (0.28–1.63), 0.383 | 0.79 (0.31–2.05), 0.632 |
| Long-term antiplatelet use | 0.53 (0.13–2.25), 0.392 | 0.74 (0.44–1.24), 0.253 | 1.20 (0.66–2.18), 0.542 | 0.63 (0.22–1.79), 0.387 | 0.42 (0.06–3.05), 0.389 | 1.35 (0.62–2.92), 0.452 | 0.48 (0.13–1.75), 0.269 |

N/A indicates estimates not calculable due to sparse data or perfect prediction

**Abbreviations:** aOR, adjusted odds ratio; CI, confidence interval

**Supplementary Table 7. Multivariable logistic regression analyses adjusted for all covariates included in the study**

| **Covariate** | **VTE OR (95% CI), p-value** | **ACS OR (95% CI), p-value** | **GIH OR (95% CI), p-value** | **ICH OR (95% CI), p-value** | **RBC OR (95% CI), p-value** | **Platelet OR (95% CI), p-value** | **Cryo/FFP OR (95% CI), p-value** |
| --- | --- | --- | --- | --- | --- | --- | --- |
| DIC | 2.94 (0.82–10.52), 0.097 | 4.39 (0.24–82.00), 0.321 | 12.41 (4.36–35.35), <0.001 | 3.88 (0.74–20.20), 0.108 | 2.86 (1.30–6.29), 0.009 | 5.67 (2.32–13.86), <0.001 | 12.70 (4.48–35.98), <0.001 |
| Age | 1.01 (0.98–1.03), 0.592 | 1.03 (0.99–1.08), 0.184 | 0.99 (0.97–1.02), 0.677 | 1.00 (0.95–1.05), 0.988 | 0.99 (0.98–1.01), 0.384 | 1.00 (0.98–1.02), 0.823 | 1.00 (0.98–1.03), 0.913 |
| Sex/Female | 0.97 (0.56–1.69), 0.920 | 1.34 (0.41–4.34), 0.629 | 0.74 (0.34–1.60), 0.441 | 0.68 (0.28–1.62), 0.382 | 1.23 (0.91–1.68), 0.186 | 0.90 (0.57–1.41), 0.636 | 1.01 (0.57–1.82), 0.963 |
| **Race/Ethnicity** | | | | | | | |
| Black | 1.58 (0.57–4.37), 0.375 | 2.37 (0.51–11.00), 0.269 | 0.33 (0.04-2.81), 0.309 | 0.54 (0.07-4.13), 0.556 | 1.36 (0.76–2.42), 0.302 | 0.65 (0.28–1.53), 0.329 | 0.80 (0.23–2.76), 0.728 |
| Hispanic | 1.86 (0.74–4.67), 0.187 | 4.00 (0.76–21.09), 0.102 | 0.67 (0.24-1.92), 0.457 | 1.11 (0.45-2.75), 0.828 | 1.34 (0.78–2.30), 0.287 | 1.52 (0.81–2.84), 0.193 | 0.79 (0.29–2.18), 0.648 |
| Asian or Pacific Islander | 0.55 (0.07–4.27), 0.563 | 1 (N/A), N/A | 0.67 (0.08-5.76), 0.714 | 1.18 (0.13-10.72), 0.882 | 1.48 (0.71–3.06), 0.296 | 1.52 (0.81–2.84), 0.193 | 0.39 (0.05–3.27), 0.383 |
| Native American | 4.75 (0.67–33.47), 0.118 | 29.25 (1.14–750.71), 0.041 | 1 (N/A), N/A | 1 (N/A), N/A | 1 (N/A), N/A | 1 (N/A), N/A | 1 (N/A), N/A |
| Other | 1.29 (0.26–6.32), 0.750 | 1 (N/A), N/A | 1.30 (0.34-4.94), 0.699 | 0.94 (0.17-5.08), 0.945 | 0.26 (0.06–1.08), 0.065 | 0.85 (0.19–3.78), 0.833 | 1.16 (0.23–5.96), 0.855 |
| CCI | 0.87 (0.37–2.06), 0.747 | 1 (N/A), N/A | 4.25 (0.80-22.52), 0.089 | 1 (N/A), N/A | 1.21 (0.68–2.12), 0.512 | 1.60 (0.82–3.12), 0.173 | 1.99 (0.60–6.60), 0.261 |
| **Insurance type** | | | | | | | |
| Medicaid | 1.04 (0.29–3.68), 0.954 | 1 (N/A), N/A | 1.71 (0.46–6.39), 0.422 | 1.54 (0.26–8.95), 0.632 | 0.84 (0.48–1.46), 0.536 | 1.54 (0.69–3.45), 0.295 | 1.47 (0.41–5.25), 0.551 |
| Private | 1.75 (0.82–3.77), 0.150 | 0.93 (0.32–2.72), 0.899 | 1.14 (0.42–3.07), 0.797 | 0.63 (0.22–1.75), 0.373 | 0.96 (0.67–1.39), 0.839 | 1.01 (0.54–1.88), 0.984 | 0.90 (0.44–1.85), 0.770 |
| Self-pay | 1 (N/A), N/A | 1 (N/A), N/A | 1 (N/A), N/A | 1 (N/A), N/A | 1.80 (0.70–4.62), 0.222 | 0.76 (0.16-3.65), 0.728 | 4.07 (1.28–12.91), 0.017 |
| Hospital teaching status/location | 1 (N/A), N/A | 1 (N/A), N/A | 1 (N/A), N/A | 1 (N/A), N/A | 1 (N/A), N/A | 1 (N/A), N/A | 1 (N/A), N/A |
| **ZIP income quartile** | | | | | | | |
| 2nd | 1.50 (0.61–3.67), 0.376 | 4.48 (0.76–26.42), 0.098 | 1.40 (0.43–4.52), 0.578 | 1.27 (0.42–3.86), 0.674 | 1.58 (0.98–2.54), 0.059 | 1.17 (0.64–2.14), 0.613 | 2.33 (0.90–6.03), 0.082 |
| 3rd | 1.41 (0.59–3.34), 0.438 | 2.80 (0.53–14.84), 0.226 | 1.44 (0.49–4.22), 0.505 | 1.15 (0.36–3.63), 0.815 | 1.76 (1.13–2.76), 0.013 | 1.32 (0.75–2.32), 0.344 | 1.83 (0.61–5.48), 0.283 |
| 4th | 0.76 (0.30–1.97), 0.576 | 2.40 (0.16–36.32), 0.529 | 0.82 (0.26–2.55), 0.730 | 0.72 (0.20–2.54), 0.609 | 1.24 (0.71–2.14), 0.446 | 1.01 (0.48–2.11), 0.975 | 1.09 (0.39–3.07), 0.871 |
| Sepsis | 9.47 (2.21–40.62), 0.002 | 1 (N/A), N/A | 8.22 (1.43–47.17), 0.018 | 1 (N/A), N/A | 2.42 (0.72–8.18), 0.153 | 3.49 (0.73–16.81), 0.118 | 1 (N/A), N/A |
| CRS | 0.92 (0.47–1.79), 0.805 | 0.31 (0.07–1.47), 0.141 | 0.87 (0.43–1.76), 0.698 | 0.76 (0.32–1.79), 0.525 | 1.19 (0.79–1.78), 0.408 | 1.55 (0.90–2.66), 0.116 | 1.94 (0.97–3.87), 0.061 |
| SARS-CoV-2 | 1 (N/A), N/A | 1 (N/A), N/A | 1 (N/A), N/A | 4.64 (1.30–16.60), 0.018 | 1.21 (0.36-4.09), 0.760 | 1.43 (0.35-5.84), 0.620 | 1 (N/A), N/A |
| Dyslipidemia | 1.07 (0.57–2.01), 0.831 | 2.33 (0.73–7.43), 0.154 | 0.81 (0.32–2.09), 0.667 | 1.10 (0.45–2.73), 0.834 | 0.84 (0.56–1.28), 0.420 | 0.80 (0.48–1.33), 0.391 | 1.39 (0.76–2.54), 0.288 |
| Hypertension | 1.07 (0.56–2.04), 0.828 | 0.99 (0.14–6.94), 0.993 | 1.11 (0.52–2.35), 0.791 | 0.74 (0.31–1.76), 0.503 | 1.22 (0.90–1.64), 0.194 | 1.38 (0.87–2.18), 0.172 | 1.38 (0.63–3.03), 0.417 |
| Atrial fibrillation | 1.08 (0.43–2.69), 0.865 | 1.51 (0.25–9.11), 0.656 | 2.55 (0.97–6.69), 0.058 | 1.12 (0.29–4.34), 0.871 | 1.11 (0.72–1.73), 0.634 | 1.62 (0.87–3.02), 0.131 | 0.97 (0.38–2.45), 0.946 |
| Long-term anticoagulant use | 2.82 (1.27–6.28), 0.011 | 0.79 (0.09–6.96), 0.831 | 0.49 (0.11–2.28), 0.364 | 0.33 (0.04–2.58), 0.288 | 0.91 (0.54–1.52), 0.709 | 0.78 (0.37–1.65), 0.520 | 0.57 (0.18–1.85), 0.353 |
| Long-term antiplatelet use | 0.85 (0.28–2.61), 0.774 | 1.07 (0.20–5.78), 0.933 | 1 (N/A), N/A | 0.52 (0.07–4.02), 0.534 | 1.12 (0.64–1.96), 0.687 | 1.01 (0.48–2.12), 0.985 | 0.81 (0.28–2.34), 0.692 |

N/A indicates estimates not calculable due to sparse data or perfect prediction

**Abbreviations:** aOR, adjusted odds ratio; CI, confidence interval

**Supplementary Table 8. Multivariable linear regression analyses adjusted for all covariates included in the study**

| **Covariate** | **LOS** β **(95% CI), p-value** | **TOTCHG** β **(95% CI), p-value** |
| --- | --- | --- |
| DIC | 17.62 (10.16–25.08), 0.001 | 523322.6 (82052.6–964592.7), 0.020 |
| Age | −0.06 (−0.12 to 0.004), 0.066 | 3902.2 (−867.0–8671.5), 0.109 |
| Sex/Female | 0.34 (−0.83–1.51), 0.573 | −39025.9 (−157116.1–79064.4), 0.517 |
| **Race/Ethnicity** | | |
| Black | −0.94 (−2.88–1.00), 0.341 | −104430.5 (−299158.9–90297.9), 0.293 |
| Hispanic | 1.01 (−0.77–2.79), 0.267 | 48148.3 (−149736.7–246033.3), 0.633 |
| Asian or Pacific Islander | 1.42 (−2.84–5.68), 0.461 | 76121.5 (−163845–316088), 0.534 |
| Native American | −6.22 (−11.23– −1.21), 0.015 | −608019.5 (−1116561– −99477.7), 0.019 |
| Other | 2.40 (−0.93–6.24), 0.219 | −25110.2 (−341391.8–291171.3), 0.876 |
| CCI | 1.90 (0.22–3.58), 0.027 | 126211.7 (−212024.6–464448), 0.464 |
| **Insurance type** | | |
| Medicaid | 2.30 (−0.43–5.02), 0.099 | 125850.7 (−92720.3–344421.6), 0.259 |
| Private | 0.17 (−1.34–1.68), 0.826 | −29709.8 (−143030.5–83611.0), 0.607 |
| Self-pay | −0.03 (−9.78–9.73), 0.996 | −407635.9 (−856989.8–41717.9), 0.075 |
| Hospital teaching status/location | 2.41 (−3.00–7.81), 0.383 | 709143.5 (270060.2–1148227), 0.002 |
| **ZIP income quartile** | | |
| 2nd | −1.59 (−3.15– −0.038), 0.045 | −36479.2 (−224403.8–151445.3), 0.704 |
| 3rd | −0.72 (−2.40–0.95), 0.397 | −79235.9 (−303432.4–144960.6), 0.488 |
| 4th | −1.06 (−2.69–0.57), 0.204 | −131373.8 (−363486.4–100738.8), 0.267 |
| Sepsis | 21.92 (9.04–34.79), 0.001 | 238107.1 (−333201.9–809416.2), 0.414 |
| CRS | −0.67 (−1.92–0.58), 0.297 | 366140.0 (188651.6–543628.4), <0.001 |
| SARS-CoV-2 | 2.26 (−2.86–7.39), 0.386 | −125272.2 (−484789.7–234245.4), 0.495 |
| Dyslipidemia | 0.96 (−0.57–2.49), 0.217 | 48054.0 (−84106.0–180214.0), 0.476 |
| Hypertension | 0.52 (−0.73–1.76), 0.411 | −13971.4 (−108406.6–80463.8), 0.772 |
| Atrial fibrillation | 2.37 (0.18–4.56), 0.034 | 49273.7 (−97931.0–196478.4), 0.512 |
| Long-term anticoagulant use | −2.68 (−4.36– −1.00), 0.002 | −21051.3 (−253177.2–211074.7), 0.859 |
| Long-term antiplatelet use | −1.76 (−3.77–0.25), 0.087 | −24821.2 (−314558.7–264916.3), 0.867 |

**Abbreviations:** β, beta coefficient; CI, confidence interval; LOS, length of stay; TOTCHG, total hospitalization charges

**Supplementary Table 9: Validation metrics of ICD-10–based outcome definitions**

| Variable | Description | Sensitivity | Specificity | PPV | Reference No. |
| --- | --- | --- | --- | --- | --- |
| AKI | Acute kidney injury | 17-57% | >98% | >90% | 1, 2, 3 |
| RRT | Renal replacement therapy | 71.8–73.9% | 99.83–99.95% | 79.9–91.6% | 4, 5 |
| RF | Respiratory failure | 12.7% to 80.8% | >95% | >90% | 6, 7 |
| MV | Mechanical ventilation use | 58.4% | 96.00% | 89.60% | 8 |
| Shock | All-cause shock | 12.7–80.8% | ~100% | 86.10% | 7, 9 |
| Vasopressor Use | Vasopressor Use | 23% | ~100% | 88% | 10, 11 |
| VTE | Acute venous thromboembolism (deep vein thrombosis and pulmonary embolism) | 75% | PE – 91.86% DVT – 95.77% | 73.3% | 12 |
| ACS | Acute coronary syndrome | 82–94% | 91–99% | 80–96%, | 13, 14 |
| GIH | Gastrointestinal hemorrhage | 65–96% | 88–99% | 71–94% | 15, 16 |
| ICH | Non-traumatic intracranial hemorrhage | 87-89% | 83% | 76-98% | 17, 18 |
| RBC | Red blood cell transfusion | 83-85% | 99-100% | ~100% | 19, 20 |
| Platelet | Platelet transfusion | N/A | N/A | N/A | N/A |
| FFP | Fresh frozen plasma transfusion | N/A | N/A | N/A | N/A |
| Cryo | Cryoprecipitate transfusion | N/A | N/A | N/A | N/A |

N/A indicates not available.

Abbreviation: PPV, positive predictive value; No, number

**References**

1. Vlasschaert ME, Bejaimal SA, Hackam DG, Quinn R, Cuerden MS, Oliver MJ, Iansavichus A, Sultan N, Mills A, Garg AX. Validity of administrative database coding for kidney disease: a systematic review. Am J Kidney Dis. 2011 Jan;57(1):29-43. doi: 10.1053/j.ajkd.2010.08.031. PMID: 21184918.
2. Esposito P, Cappadona F, Marengo M, Fiorentino M, Fabbrini P, Quercia AD, Garzotto F, Castellano G, Cantaluppi V, Viazzi F. Recognition patterns of acute kidney injury in hospitalized patients. Clin Kidney J. 2024 Jul 22;17(8):sfae231. doi: 10.1093/ckj/sfae231. PMID: 39157067; PMCID: PMC11328729.
3. Tomlinson LA, Riding AM, Payne RA, Abel GA, Tomson CR, Wilkinson IB, Roland MO, Chaudhry AN. The accuracy of diagnostic coding for acute kidney injury in England - a single centre study. BMC Nephrol. 2013 Mar 13;14:58. doi: 10.1186/1471-2369-14-58. PMID: 23496869; PMCID: PMC3599863.
4. Gao C, Xu Y, Mehta S, Sang Y, Flaherty C, Surapaneni A, Pandit K, Chang AR, Green JA, Grams ME, Shin JI. Validation of an Algorithm to Identify End-Stage Kidney Disease in Electronic Health Records Data. Am J Kidney Dis. 2025 Aug;86(2):212-221.e1. doi: 10.1053/j.ajkd.2025.03.021. Epub 2025 May 15. PMID: 40381931; PMCID: PMC12286722.
5. Taneja C, Berger A, Inglese GW, Lamerato L, Sloand JA, Wolff GG, Sheehan M, Oster G. Can dialysis patients be accurately identified using healthcare claims data? Perit Dial Int. 2014 Sep-Oct;34(6):643-51. doi: 10.3747/pdi.2012.00328. Epub 2014 Feb 4. PMID: 24497600; PMCID: PMC4164409.
6. Quan H, Li B, Saunders LD, Parsons GA, Nilsson CI, Alibhai A, Ghali WA; IMECCHI Investigators. Assessing validity of ICD-9-CM and ICD-10 administrative data in recording clinical conditions in a unique dually coded database. Health Serv Res. 2008 Aug;43(4):1424-41. doi: 10.1111/j.1475-6773.2007.00822.x. PMID: 18756617; PMCID: PMC2517283.
7. Plummer AL. International Classification of Diseases, Tenth Revision, Clinical Modification for the Pulmonary, Critical Care, and Sleep Physician. Chest. 2015 Nov;148(5):1353-1360. doi: 10.1378/chest.15-0487. PMID: 26110875.
8. Wunsch H, Kramer A, Gershengorn HB. Validation of Intensive Care and Mechanical Ventilation Codes in Medicare Data. Crit Care Med. 2017 Jul;45(7):e711-e714. doi: 10.1097/CCM.0000000000002316. PMID: 28403118; PMCID: PMC6557134.
9. Wang Y, Song Y, Siu R, et al. Validation of 13 102 International Classification of Diseases, Tenth Revision, Clinical Modification codes using a large language model-based system. *J Am Med Inform Assoc.* 2026 Feb 11. doi:10.1093/jamia/ocag008.
10. Garland A, Marrie RA, Wunsch H, Yogendran M, Chateau D. Accuracy of Administrative Hospital Data to Identify Use of Life Support Modalities. A Canadian Study. Ann Am Thorac Soc. 2020 Feb;17(2):229-235. doi: 10.1513/AnnalsATS.201902-106OC. PMID: 32003608.
11. Lauridsen MD, Gammelager H, Schmidt M, Nielsen H, Christiansen CF. Positive predictive value of International Classification of Diseases, 10th revision, diagnosis codes for cardiogenic, hypovolemic, and septic shock in the Danish National Patient Registry. BMC Med Res Methodol. 2015 Mar 20;15:23. doi: 10.1186/s12874-015-0013-2. PMID: 25888061; PMCID: PMC4373092.
12. Alotaibi GS, Wu C, Senthilselvan A, McMurtry MS. The validity of ICD codes coupled with imaging procedure codes for identifying acute venous thromboembolism using administrative data. Vasc Med. 2015 Aug;20(4):364-8. doi: 10.1177/1358863X15573839. Epub 2015 Apr 1. PMID: 25834115.
13. Slezak J, Bruxvoort KJ, Sy LS, Ackerson BK, Shen AY, Lee MS, Gupta N, Qian L, Qiu S, Solano Z, Reynolds K. Validation of ICD-10 Diagnosis Codes for Identification of Acute Myocardial Infarction From a US Integrated Healthcare System. Pharmacoepidemiol Drug Saf. 2025 Jul;34(7):e70179. doi: 10.1002/pds.70179. PMID: 40552766; PMCID: PMC12186587.
14. Tsai TY, Lin JF, Tu YK, Lee JH, Hsiao YT, Sung SF, Tsai MJ. Validation of ICD-10-CM Diagnostic Codes for Identifying Patients with ST-Elevation and Non-ST-Elevation Myocardial Infarction in a National Health Insurance Claims Database. Clin Epidemiol. 2023 Oct 17;15:1027-1039. doi: 10.2147/CLEP.S431231. PMID: 37868152; PMCID: PMC10590151.
15. Oger E, Botrel MA, Juchault C, Bouget J. Sensitivity and specificity of an algorithm based on medico-administrative data to identify hospitalized patients with major bleeding presenting to an emergency department. BMC Med Res Methodol. 2019 Oct 18;19(1):194. doi: 10.1186/s12874-019-0841-6. PMID: 31627721; PMCID: PMC6798331.
16. Thaarup M, Nielsen PB, Olesen AE, Bitsch Poulsen M, Larsen TB, Wittström F, Overvad TF. Positive Predictive Value of Non-Traumatic Bleeding Diagnoses in the Danish National Patient Register. Clin Epidemiol. 2023 Apr 28;15:493-502. doi: 10.2147/CLEP.S400834. PMID: 37144211; PMCID: PMC10153536.
17. Sangal RB, Fodeh S, Taylor A, Rothenberg C, Finn EB, Sheth K, Matouk C, Ulrich A, Parwani V, Sather J, Venkatesh A. Identification of Patients with Nontraumatic Intracranial Hemorrhage Using Administrative Claims Data. J Stroke Cerebrovasc Dis. 2020 Dec;29(12):105306. doi: 10.1016/j.jstrokecerebrovasdis.2020.105306. Epub 2020 Oct 15. PMID: 33070110; PMCID: PMC7686163.
18. Hsieh MT, Huang KC, Hsieh CY, Tsai TT, Chen LC, Sung SF. Validation of ICD-10-CM Diagnosis Codes for Identification of Patients with Acute Hemorrhagic Stroke in a National Health Insurance Claims Database. Clin Epidemiol. 2021 Jan 14;13:43-51. doi: 10.2147/CLEP.S288518. PMID: 33469381; PMCID: PMC7813455.
19. Segal JB, Ness PM, Powe NR. Validating billing data for RBC transfusions: a brief report. Transfusion. 2001 Apr;41(4):530-3. doi: 10.1046/j.1537-2995.2001.41040530.x. PMID: 11316905.
20. Patterson JA, Francis S, Ford JB. Assessing the Accuracy of Reporting of Maternal Red Blood Cell Transfusion at Birth Reported in Routinely Collected Hospital Data. Matern Child Health J. 2016 Sep;20(9):1878-85. doi: 10.1007/s10995-016-1992-2. PMID: 27013516.
